# The Effect of Educating Health Promotion Strategies on Self-Care Self-Efficacy in Patients Undergoing Kidney Transplantation: A Double Blind Randomized Trial

**DOI:** 10.5812/nms.11874

**Published:** 2013-12-09

**Authors:** Fateme Soltannezhad, Zahra Farsi, Masoud Jabari Moroei

**Affiliations:** 1 Department of Medical-Surgical, AJA University of Medical Sciences, Tehran, IR Iran

**Keywords:** Education, Kidney, Transplantation, Self-Care, Self-Efficacy, Health Promotion

## Abstract

**Background:**

Self-care self-efficacy in patients with end stage renal disease, waiting for kidney transplantation, probably decreases due to facing new conditions and side effects of treatment.

**Objectives:**

The current study was conducted to investigate the effect of educating health promotion strategies on self-care self-efficacy in patients undergoing kidney transplantation.

**Patients and Methods:**

A double blind randomized clinical trial was conducted on 52 patients undergoing kidney transplantation in Baqiyatallah Hospital in 2012. Patients were randomly assigned into intervention and control groups. The questionnaire of Strategies Used by People to Promote Health (SUPPH) was employed to measure self-care self-efficacy. At first, the two groups filled the questionnaire. Then, the intervention group was trained regarding health promotion strategies within 4 sessions before the transplantation. The control group was trained according to routine protocol of the transplantation unit. Then, the two groups were followed up for two months, and reassessed at the end of the first and second months after the transplantation. The data were analyzed by descriptive and analytic statistics including independent samples T test, Chi square and repeated measures ANOVA.

**Results:**

In the intervention group, the mean of total self-care self-efficacy was 106.96 ± 25.1 at first, and changed to 135.81 ± 9.65 and 111.19 ± 12.45 after the first and second post-test respectively (P = 0.001). In the control group, the mean of total self-care self-efficacy was 112.73 ± 14.33 at first, and changed to 118.58 ± 17.59 and 108.73 ± 15.93 after the first and second post-test respectively (P = 0.001). Significant differences were observed between the two groups in the first post-test regarding total score of self-care self-efficacy (P = 0.001) and dimensions of reduction of stress (P = 0.001), enjoying life (P = 0.01), and coping (P = 0.001). The mean scores of the intervention group were higher than those of the controls in all domains of self-care self-efficacy in the second post-test. However, the difference was only significant in decision-making dimension (P = 0.04).

**Conclusions:**

Educating health promotion strategies was effective in improving self-care self-efficacy in patients undergoing kidney transplantation. Establishment of a holistic caring program is suggested to integrate the pre-transplantation educations with a continual post discharge follow-up.

## 1. Background

End stage renal disease (ESRD) is a chronic and progressive deterioration in renal function in which the body’s ability to maintain metabolic, fluid, and electrolyte balance fails (1). In 2009, at least 116395 new cases of ESRD were diagnosed in the United States. In 2011, more than 31 million people (about 10% of the population) had ESRD in the US, and approximately 14,000 of them underwent kidney transplants (2). In Iran, the annual number of kidney transplantations increased from fewer than 100 in 1986 (3) to more than 2000 cases in 2012 (4). Kidney transplantation is accounted as the greatest progresses in modern medicine. It is the best alternative treatment in patients with ESRD, which increases quality of life (QOL) and hope, and decreases health care costs in comparison with hemodialysis (1, 2). In spite of the progresses in transplantation, there are still numerous problems (1). Despite the improvement of QOL in recipients, mortality rate is four times more in comparison with general population in the first year after the transplantation (5). Although advancements in surgical techniques and immunosuppressive drugs have improved the process of organ transplantation, patients still tolerate problems in drugs management and their unpleasant side effects, and fear for rejection of the transplanted organ that causes refusal of treatments and disobedience of health care recommendations, which in turn reduce patients’ ability to cope effectively (1). Besides, prolonged use of immunosuppressive drugs and surgery complications create many side effects (6-8), which cause severe psychological tension, anxiety and uncertainty about future, and maladjustment for patients (9). Studies have shown that patients undergoing transplantation experience depression, and their life quality decreases (10, 11). These complications result in decreases of self-efficiency and self-caring behaviors (10, 11) which would intensify their depression and stress (12). A study showed that the need for renal replacement therapy can diminish patients’ self-efficacy, which is the belief about ability to control the environment and life circumstances (13). The concept of self-efficacy was first introduced by Bandura and was defined as ability to do a particular activity and expecting ability to do a determined behavior successfully (11). Within this theory, self-care self-efficacy is defined as a person’s confidence in being able to perform relevant self-care behaviors in a particular situation (11). Some studies showed that self-efficacy has positive effects on problem solving, patient–provider partnership, self-care behavior, and self-management. Self-efficacy and self-care behaviors affect the mental health component of QOL (14). Also, some evidences show that increase in self-care self-efficacy causes more adjustment, reduces psychological and physical symptoms, and improves self-care behaviors, life satisfaction and QOL (15). The evidence indicates that self-care self-efficiency probably changes through implementing health promotion strategies (13). Health promotion strategies focus on appropriate caring of individuals and disease prevention. These strategies influence individuals' health through prevention levels (16). Screening of cancers, control of risk factors of cardiovascular diseases, convenient diet, doing exercise, and controlling stress and infection are some of the health promotion strategies in patients undergoing kidney transplantation (17). Evidence shows that educating health promotion strategies can reduce stress and increase self-efficacy, improve self-caring, and reduce cost (11). Also, training patients is the cornerstone of nursing. Elis Lev has designed a questionnaire to measure individuals’ confidence of self-care self-efficacy and implementing health promotion strategies named as Strategies Used by People to Promote Health (SUPPH) (18, 19). She used this tool to measure self-efficacy in patients with cancer and patients undergoing hemodialysis, and reported that educating health promotion strategies to patients could increase patients and their families’ satisfaction from caring quality, improve QOL, increase self-efficacy, and confidence of care continuation, reduce anxiety and disease complications, enhance patients’ participation in care plan and their independence in performing daily activities, and also would decrease patients’ costs and hospital stay (11). Azizi-Fini et al. reported that educating health promotion strategies increases self-efficacy, and then enhances coping with disease, reduces stress, and improves decision making in patients candidate for bone marrow transplantation. However, they reported that this education could not significantly increase the patients’ life enjoyment (11). Some other studies also reported that teaching health promotion strategies did not significantly affect some dimensions of self-efficacy (20, 21). Due to the mentioned conflicts on the impact of health-promotion strategies education, and limited studies on educating health promotion strategies in patients undergoing kidney transplantation, there is a question about the effect of health promotion strategies on self-care self-efficacy of patients undergoing kidney transplantation. 

## 2. Objectives

The current study aimed to investigate the effect of educating health promotion strategies on self-care self-efficacy in patients undergoing kidney transplantation.

## 3. Patients and Methods

**Figure 1.**
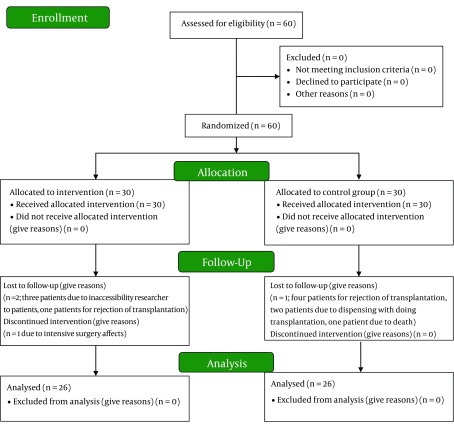
The Study Design

A double blind randomized clinical trial was conducted on 60 patients undergoing kidney transplantation. The study population was patients undergoing kidney transplantation referring to Baqiyatallah Hospital in Tehran, Iran from September to March 2012. Patients were recruited through convenience sampling method. Inclusion criteria were as follows: aged 18 years or over, be able to understand, speak and write in Persian, and have adequate visional, auditory, speaking and psychological abilities to participate in the study. Exclusion criteria were as follows: rejection of transplantation, refusal of recipient to undergo transplantation, and severe complications of transplantation. Considering type one error (α = 0.05), type II error (β = 0.1), and according to mean and standard deviation in a previous study (11), and using sampling formula (22)

n = (Z_1 – α/2 _+ Z_1 - β_) ^2 ^(δ_1 _^2 ^+ δ_2 _^2 ^)/(µ_1 _- µ_2 _) ^2 ^= (1.96 + 1.28) ^2 ^(10.32 ^2 ^+ 9.38 ^2^)/(14.83 -4.93) ^2^,

the number of subjects in each group was estimated to be 21 patients. Sixty patients were finally recruited in the research, 30 in each group, by considering a possible attrition. Despite the fact that the researchers tried to prevent attritions through attending in the field and telephone follow up, some of the patients did not complete the study. During the research, four patients in the control group and four patients in the intervention group (one patient for death, two patients for rejection of transplantation, one patient due to major surgical complications, two patients for inaccessibility by the researcher, and two patients due to declining to do transplantation) were excluded from the study, and the research was finalized with 52 subjects (Figure 1). The patients and analyzer were blinded to group assignments into case and control, but the assessor (F.S) was not blinded. 

A two part questionnaire was used for data collection. The first part included six questions on patients’ demographic characteristics including age, sex, marital status, education, income, and the type of donor. The second part was SUPPH questionnaire. The primary version of this questionnaire had 29 items. It is a self-report tool to measure self-care self-efficacy, and it has been used in research and practice settings (11). All items of the SUPPH are answered in a five-choice Likert scale ranging from too high (= 5) to too low (= 1). This questionnaire evaluates four dimensions of coping, reduction of stress, decision making, and enjoying life. The questionnaire evaluates the amount of individuals' certainty about doing these dimensions. The questionnaire was translated into Persian, and was validated in a previous study (11). In the current research, facial and content validities of the questionnaire were confirmed again by ten experts in AJA, Baqiyatallah, Tehran, and Shahid Beheshti Universities of Medical Sciences. In the process of content validity, two items (I am confident that I can overcome difficulties with faith in Allah, I am confident that I am strong) were added to cover all dimensions in the final 29-item version of the questionnaire. Then, the scores of this new version were ranged from 31 to 155. Reliability was confirmed by test-retest method. Ten patients undergoing kidney transplantation answered the questionnaire twice in two weeks interval. Then, correlation coefficient was calculated as 0.95, and Cronbach's Alpha coefficient was 0.95. The subjects were reviewed three times, once before (1-3 days), and twice after the transplantation. All the three occasions of the evaluations were conducted in a private room in the transplantation unit. After completion of pretest in the two groups, each patient in intervention group was trained within four 30-minute sessions by the researcher (F.S) about stress management, coping strategies, nutrition, exercise, medication consuming, and self-caring after the operation, and common complications in kidney recipients. All educational sessions were conducted prior to the transplantation, individually and face to face. The patients in the two groups were followed up after the operation in the transplantation unit. Also, an educational booklet and the researcher's telephone number were given to the intervention group members for probable questions. An educational pamphlet was also delivered to the family members of the intervention group. The control group was trained according to the routine training protocol by the nurses of transplantation unit. The first and second stages of posttest were performed at the end of the first and second months after the transplantation in the two groups. The first posttest was conducted in the transplant unit, and the second posttest was performed by the researcher through telephone interview. 

### 3.1. Ethical Considerations

The Research Council and the Research Ethics committee in the AJA University of Medical Sciences approved this study. Data collection was performed after explaining the research objectives, and obtaining informed consent from the participants. The researchers bounded themselves to observe the ethical issues based on the Helsinki Declaration (23). All patients were assured of obscurity and confidentiality of their personal information, and the right to refuse participation or withdraw from the study at any time. Also the necessary permissions were sought from the hospital authorities and the transplantation unit.

### 3.2. Data Analysis

SPSS software version 15 was employed to analyze the data. The data was analyzed by descriptive and analytic statistics including Independent-Samples T test, Chi square, and repeated measures analysis of variance.

## 4. Results

In total, 52 patients completed the study. The mean age of patients was 37.88 ± 9.72; 71.2% of the patients were male; 15.4% were single, 75% were married, and 9.6% were divorced. About 23% of the participants had academic education, and 76.9% of the patients had high school diploma or lower education. In total, 59.6% of the patients received kidney from a live donor, and 40.4% from a cadaver. No statistically significant differences were observed between the two groups regarding demographic variables (P > 0.05) (Table 1). The mean of self-care self-efficacy and its dimensions in each of the two groups in three stages of pretest, first and second posttests are shown in Table 2. 

**Table 1.** Comparison of Demographic Variables in the Intervention and Control Groups

Variables	Intervention Group	Control Group	Test	P value
**Age, No. (Mean ± SD), y**	26 (38.12 ± 9.32)	26 (37.65 ± 10.3)	t-Test	0.71
**Gender, No. (%)**			Fisher’s Exact Test	0.50
Female	7 (46.7)	8 (53.3)		
Male	19 (51.4)	18 (48.6)		
**Marital Status, No. (%)**			χ ^2^	0.23
Single	3 (37.5)	5 (62.5)		
Married	22 (56.4)	17 (43.6)		
Widow	1 (20.0)	4 (80.0)		
**Education, No. (%)**			χ ^2^	0.13
Primary	11 (45.8)	13 (54.2)		
Diploma	7 (43.8)	9 (56.3)		
Academic degree	8 (66.7)	4 (33.3)		
**Income, IRR, No. (%)**			Fisher’s Exact Test	0.99
<5000000	16 (48.5)	17 (51.5)		
>5000000	10 (52.63)	9 (47.36)		
**Type of donor, No. (%)**			Fisher’s Exact Test	0.50
Alive	16 (51.6)	15 (48.4)		
Cadaver	10 (47.6)	11 (52.4)		

**Table 2.** The Means and Standard Deviation of Self-Efficacy in Three Stages in the Intervention and Control Groups

Dimensions of self-care self-efficacy	Intervention group, Mean(SD)	Control group, Mean(SD)	P value
**Pre-test**			
Reduction of stress	15.90 (4.58)	16.77 (3.5)	0.41
Enjoying life	17.69 (4.28)	18.70 (2.60)	0.28
Decision making	8.10 (1.73)	8.60 (1.35)	0.21
Coping	66.00 (14.70)	67.93 (8.94)	0.54
Self-care self-efficacy	106.96 (25.1)	112.73 (14.33)	0.31
**Post-test 1**			
Reduction of stress	21.27 (2.03)	17.82 (3.30)	0.001
Enjoying life	22.15 (2.09)	19.86 (2.86)	0.01
Decision making	9.31 (1.03)	9.32 (1.02)	0.96
Coping	83.08 (6.06)	70.25 (11.45)	0.001
Self-care self-efficacy	135.81 (9.65)	118.58 (17.59)	0.001
**Post-test 2**			
Reduction of stress	17.13 (2.36)	16.12 (3.20)	0.22
Enjoying life	16.85 (2.72)	16.81 (2.77)	0.96
Decision making	9.88 (0.44)	9.27 (1.34)	0.04
Coping	64.64 (7.77)	63.50 (9.94)	0.65
Self-care self-efficacy	111.19 (12.45)	108.73 (15.93)	0.53

Independent sample t-test was used, and no significant difference was observed between the two groups in pretest in the total score of self-care self-efficacy and its dimensions. Also significant differences were observed between the two groups in the first posttest regarding the total score of self-care self-efficacy (P = 0.001), and dimensions of reduction of stress (P = 0.001), enjoying life (P = 0.01), and coping (P = 0.001), in a way that the scores of intervention group was higher than those of the control group. The mean scores of the intervention group were higher than those of the controls in all domains of self-care self-efficacy in the second post-test. However, the difference was only significant in decision-making (P = 0.04) (Table 2). The results of repeated measures ANOVA test showed that total score of self-care self-efficacy of the patients in intervention group in the pretest, one month and two months after the transplantation were significantly different (green house- Geisser, P = 0.001), in a way that the mean scores of pretest and one month after the transplantation, and also the mean scores of self-care self-efficacy in the first and second months post-transplantation were significantly different (Bonferroni, P = 0.001). Also, Repeated Measures ANOVA showed that total scores of self-care self-efficacy in the control group were significantly different in the three stages (Sphericity Assumed, P = 0.001), in a way that a significant difference was observed between the mean scores of self-care self-efficacy at pretest and one month after the transplantation (P = 0.039). Also the mean scores of self-care self-efficacy at the first and second months post-transplantation were significantly different (Bonferroni, P = 0.001). However, as Table 3 shows, the mean of changes in self-care self-efficacy scores were considerably higher in the intervention group (Table 3). 

**Table 3.** The Mean Difference of Total Score of Self-Care Self-Efficacy in the Two Groups

	Time	Mean Difference	Standard Error	P value
**Intervention Group**				
Pre-test	Post-test 1	-28.85	3.78	0.001
Pre-test	Post-test 2	-4.23	4.38	1.0
Post-test 1	Post-test 2	24.62	2.16	0.001
**Control Group**				
Pre-test	Post-test 1	-5.85	2.19	0.039
Pre-test	Post-test 2	4.0	2.45	0.347
Post-test 1	Post-test 2	9.85	2.11	0.001

## 5. Discussion 

This research was conducted to investigate the effect of educating health promotion strategies on self-care self-efficacy of patients undergoing kidney transplantation. The results of the current research showed that educating health promotion strategies in patients undergoing kidney transplantation was effective on all dimensions of self-care self-efficacy except for decision-making. However, the condition was reverse in the second post-test. Some of the current findings are in line with the results of some previous studies. For instance, in a study conducted by, Azizi-Fini et al. on patients undergoing bone marrow transplantation it was reported that training health promotion strategies increased self-care self-efficacy regarding coping and stress reduction one month after the transplantation (11). However, the findings of the current study are inconsistent with the results of Azizi-Fini et al., regarding decision-making and enjoying life. Taghdisi et al. have also reported that training health promotion strategies could increase the mean score of self-care self-efficacy regarding coping and QOL in patients with cancer (24). Baljani et al. also studied the effects of a nursing intervention on improving self-efficacy and reducing cardiovascular risk factors in patients with cardiovascular diseases. They also showed that overall self-efficacy score was significantly improved one month after the education (25). Studies showed that stressful life events could have a negative effect on self-efficacy in recipients of transplantation (9-11). The recipients of kidney transplantation are very stressful in the first months after the transplantation due to facing new conditions and problems (such as disease problems, drugs side effects, immune-suppression, fear of rejection , being isolated, losing job, and economic problems) (9). In the current study, the mean score of decision making was relatively stable in all stages; while, the other dimensions increased in the first post-test, and then decreased in the second post-test. It seems that educating health promotion strategies has a little or delayed effect on the dimension of decision-making; while, it has a transient positive effect on other dimensions of self-care self-efficacy. Probably, decreasing social and affective supports over time were influential on decreasing the scores of these dimensions. The social context has shown to have a large influence on decision making. The evidence indicated that the effects of the social context on decision making can be both positive and negative (26). In the current research, both the intervention and control groups showed an increase in the scores of all dimensions of self-care self-efficacy; although, the scores of the intervention group were higher than those of the control group in all post-testes. This finding indicates that both the routine educations and the intervention in the current study have positive effects on the patients’ self-care self-efficacy; although, the intervention was to some extent more effective; however, the decrease in the mean scores of the second post-test shows that both the routine and the new intervention need to be complemented with continual post discharge follow-ups to be more effective. In conclusion, the findings of this study showed that educating health promotion strategies was effective in improving self-care self-efficacy in patients undergoing kidney transplantation. The feeling of self-efficacy may improve the self-care behaviors in transplantation recipients. However, the results showed the need for a holistic caring program that integrates pre transplantation educations with a continual post discharge follow-up system. There were some limitations in the current study including the small sample size and the short period of study. Therefore, it is suggested to conduct further studies with larger sample size and in longer follow-up period after hospital discharge. Also, studies on the effect of educating health promotion strategies through tele-nursing systems on self-care self-efficacy in patients undergoing kidney transplantation are recommended.
